# Effects of climate change on fungal infections

**DOI:** 10.1371/journal.ppat.1012219

**Published:** 2024-05-30

**Authors:** Samantha L. Williams, Mitsuru Toda, Tom Chiller, Joan M. Brunkard, Anastasia P. Litvintseva

**Affiliations:** 1 Mycotic Diseases Branch, Centers for Disease Control and Prevention, Atlanta, Georgia, United States of America; 2 Division of Foodborne, Waterborne, and Environmental Diseases, Centers for Disease Control and Prevention, Atlanta, Georgia, United States of America; Vallabhbhai Patel Chest Institute, INDIA

Climate change significantly impacts atmospheric, ecological, agricultural, and societal systems. Documented increases in global temperature, extreme precipitation, and the frequency and intensity of severe weather events have been linked to a variety of adverse health outcomes, and conditions are expected to worsen [1]. Fungi are particularly susceptible to the effects of climate change because the highest diversity and biomass of fungi are found in the top layer of soil, at the forefront of environmental changes. Fungal diseases cause a wide spectrum of illness, ranging from mild skin and mucosal infections to severe respiratory illness and life-threatening disseminated disease. Evidence suggests that evolving weather patterns have contributed to expanded geographic ranges of endemic fungi, emergence of new pathogens, and increased antifungal resistance [2,3]. This review presents an introduction to and discussion of some of the most important potential climate-related mechanisms associated with the proliferation of pathogenic fungi and associated fungal diseases (Fig 1) that could impact human and animal health.

**Fig 1:**
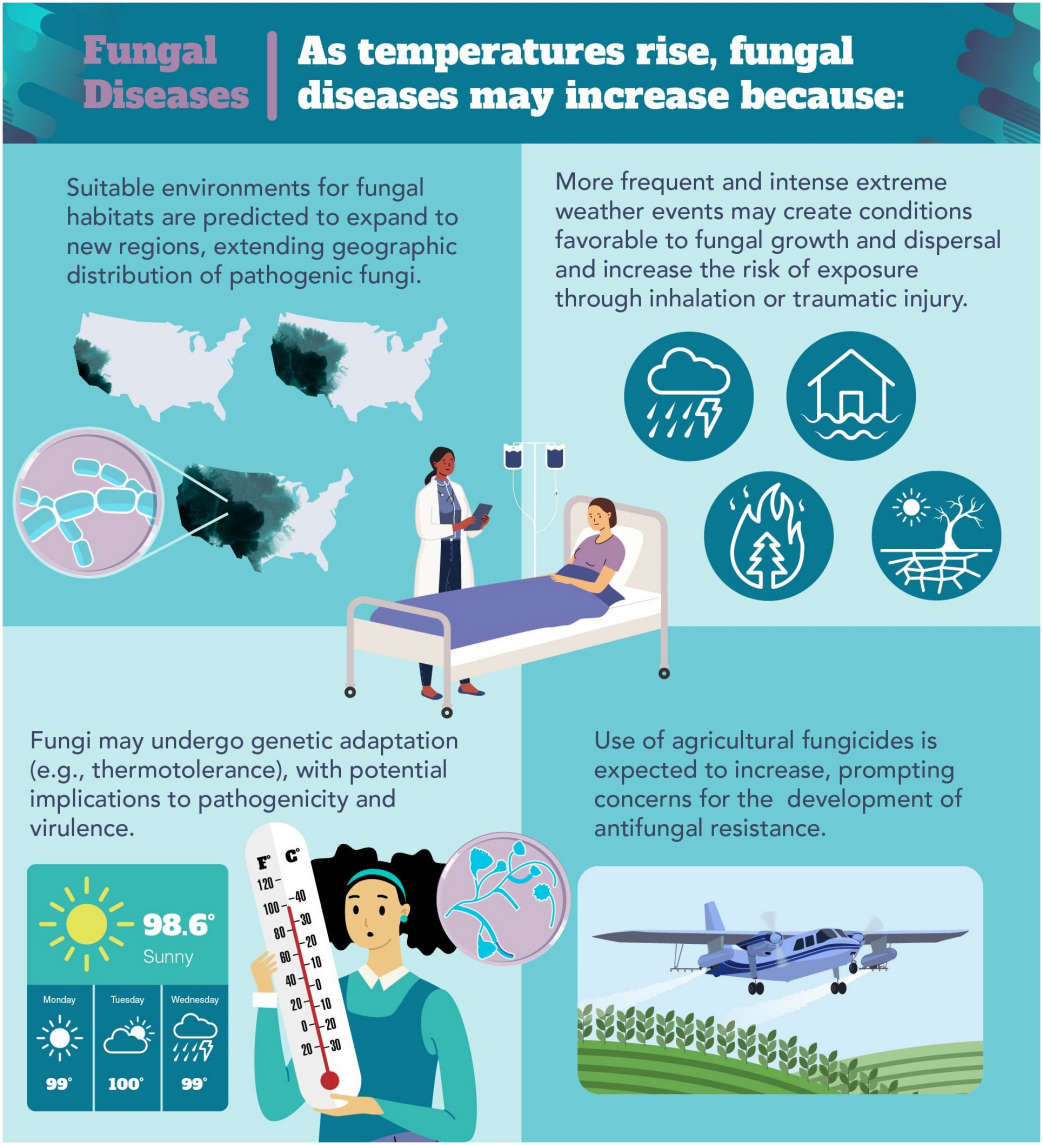
Potential effects of climate change on pathogenic fungi and fungal diseases.

## Expanding geographic distribution of pathogenic fungi

The known geographical distribution of endemic fungal diseases is expanding. For example, fungi from genus *Coccidioides* that cause coccidioidomycosis have historically been found in soil in hot and dry areas in the southwestern United States and parts of Central and South America. Because *Coccidioides* spp. are affected by rainfall and drought cycles, warming temperatures and changing precipitation patterns are extending areas for fungal growth as well as subsequent dispersal and aerosolization [4]. The fungus has now been detected as far north as Washington [5]. Climate niche modeling based on rising temperatures and rainfall dynamics predicts that *Coccidioides* could extend as far north and east as Minnesota by the end of the century [6].

In addition to temperature and precipitation, habitat suitability for fungi is influenced by soil characteristics such as pH levels, minerals, and organic content, all of which are impacted by climate change. Suitability maps for the fungus *Histoplasma*, the causative agent of histoplasmosis, showed that favorable soil environments have expanded beyond traditionally recognized areas in the Ohio and Mississippi River Valleys [7,8].

Fungi from *Cryptococcus gattii* species complex that cause cryptococcosis were restricted to tropical or subtropical climates until the 1990s, when strains of *C*. *gattii* VGII molecular type were detected in the US Pacific Northwest and British Columbia in Canada, indicating a potential shift in the ecological niche [9]. *C*. *gattii* currently lives along the Mediterranean coast, and niche modeling based on temperature and precipitation predicts that the distribution will extend toward inland regions of Europe in the next decade [10].

While climate change can directly impact fungal habitat suitability, its effects on wildlife migration patterns may also influence the geographic spread of certain fungal pathogens. Spatial movement or relocation of birds and bats, which help spread *Histoplasma* and *Cryptococcus neoformans*, and small mammals (e.g., rodents), which may serve as reservoirs for *Coccidioides* and other pathogens, can potentially expand areas where these fungi live in the environment, though the extent to which climate change has altered migratory trends is unclear [11–13].

## Impact of extreme weather on fungal growth, dispersal, and disease transmission

Scientists predict that the frequency and intensity of extreme weather events will continue to increase as average global temperatures rise [1]. Severe weather can produce both immediate and long-lasting effects on fungal habitat and risk of exposure and subsequent infection. Environmental disruptions from severe weather events such as dust storms, tornadoes, and wildfires can aerosolize fungal spores, increasing risk of airborne exposure [14,15]. In the southwestern US, the incidence of coccidioidomycosis increased while the number of dust storms doubled from 1988 to 2011 [16]. Although the consistency and extent of the association between dust storms and coccidioidomycosis is unclear, many investigators agree that dust storms pose a risk of *Coccidioides* infection and can transport arthroconidia to new locations [17].

The intensity of hurricanes is expected to increase [1]; flooding from heavy precipitation results in excessive moisture suitable for mold growth, particularly in indoor settings. Mold exposure can lead to a wide range of health effects, including upper respiratory tract symptoms; it may also lead to invasive infection among immunocompromised populations [18–22].

Inhalation of and cutaneous exposure to fungal spores are the most common means of disaster-related fungal infections. Flooding and drowning or near-drowning events increase the risk of fungal spore aspiration or cutaneous exposure to fungal-contaminated water [14]. Skin and soft tissue fungal infections may occur postdisaster, particularly if wounds are exposed to water, soil, or debris containing infectious agents. For example, a cluster of 13 necrotizing mucormycosis cases was detected following a 2011 tornado in Joplin, Missouri, and infection was associated with penetrating trauma [15]. Disasters may also compound infection risk if physical damages (e.g., power outages, building destruction) impede access to healthcare services to properly treat wounds and injuries.

Climate refugees, people displaced due to climate change, are often subjected to overcrowded, poor living conditions in hot and humid climates. These conditions are ideal for dermatophyte transmission through direct contact with affected people, animals, or through fomites [23]. There is growing concern regarding the spread of dermatophytes as emerging pathogens, such as *Trichophyton indotineae*, which can cause extensive skin lesions and have developed resistance to antifungal treatments [24].

### Evolutionary traits as a potential result of climate change?

Of nearly 144,000 species of fungi described, less than a few hundred are capable of infecting humans, and only a handful can infect people without underlying immunocompromising conditions [25]. It is hypothesized that this limited ability to infect humans is in part due to the inability of most fungi to survive at mammalian and some avian body temperatures (37 °C/98.6 °F and 40 °C/104 °F, respectively) [26]. However, rising temperatures may cause more fungal species to become pathogenic to humans as they adapt to live and replicate at higher heat, narrowing the thermal restrictive barrier between ambient and human body temperatures [27].

The multidrug-resistant yeast *Candida auris* is the first fungal pathogen proposed to have emerged as a result of adaptation to climate change [28]. Some researchers posit that global warming contributed to the simultaneous emergence of distinct clades of the species on 3 separate continents from 2012 to 2015 based on the fact that *C*. *auris* can grow at higher temperatures compared with closely related species [28]. This suggests that its acquisition of thermal tolerance and consequent transition from environmental fungus to human pathogen may have been relatively recent [28]. Concern is growing that other fungi may similarly adapt.

Recent studies found that heat stress was associated with accelerated genetic mutations of the fungal pathogen *Cryptococcus deneoformans* [29,30]. Under laboratory conditions, temperature increases promoted resistance to antifungal drugs in vitro due to transposon mobilization [30], and transposable DNA elements or “jumping genes” demonstrated 5 times more movement at 37 °C compared to 30 °C [29]. Genetic changes may contribute to greater thermotolerance, virulence, or drug resistance, although further study is needed to better understand the effects of heat-stimulated mutations and their relation to pathogenic characteristics.

### Indirect effect of global warming on antifungal resistance

Healthcare providers rely on just 3 main classes of antifungal medications (azoles, echinocandins, andpolyenes) to treat systemic fungal infections, limiting clinical options when first-line treatment fails. Strains of pathogens such as *Aspergillus fumigatus*, *C*. *auris*, and others showing resistance to one or more classes of antifungal drugs have been detected worldwide, signaling a global health threat.

Although the mechanisms of antifungal resistance are multifaceted, there is evidence that agricultural fungicides played a key role in the development of azole-resistant *A*. *fumigatus*, given their chemical similarity to antifungal medications used in clinical care. Inhalation of resistant strains from the environment can result in human infections resistant to antifungal treatment. Use of triazole fungicides in the US increased 4-fold from 2006 to 2016, and trends in azole fungicide use correlated with the sharp increase of azole-resistant *A*. *fumigatus* infections in humans [31]. Fungicide use is expected to grow as a result of climate change and the ensuing need for more concentrated and frequent applications to compensate for productivity loss due extreme weather [32–34]. This could lead soil fungi, some of which are opportunistic human pathogens, to develop and select for resistance to fungicides.

Mechanisms of cross-resistance between agricultural and clinical antifungals have been described for medications other than azoles, including a novel antifungal medication, olorofim, which is currently undergoing clinical trials with initial results demonstrating high potency against azole-resistant *A*. *fumigatus* and other difficult-to-treat fungal infections. At the same time, a novel fungicide with the same mode of action has already been approved for agricultural use, raising serious concerns about development of resistance to olorofim in the environment [3]. Growth inhibition studies showed that in vitro exposure of the fungicide to *A*. *fumigatus* can select for strains that are resistant to olorofim [35]. Similarly, fosmanogepix, an antifungal therapy in clinical trials for treatment of invasive fungal infections caused by *Candida*, *Aspergillus*, and other rare molds targets the same enzyme as another in-development fungicide, which may increase the risk of cross-resistance [3].

### Conclusions and future perspectives

Fungi are environmental organisms affected by shifts in climate over time, though the exact impact of these changes on fungal pathogens is not well understood and can be challenging to distinguish from other factors. While the potential effects of climate change have been studied for certain fungi, such as *Coccidioides*, the impact on other mycoses is less clear. Fungal infections that typically occur in tropical or subtropical climates, such as chromoblastomycosis, paracoccidioidomycosis, and eumycetoma, may experience a similar expansion in geographic distribution, but existing data are limited. Similarly, more frequent rainfall could lead to increased incidence of talaromycosis, which has been shown to peak during rainy seasons [36,37].

Notably, the risk of fungal infection may be exacerbated for certain populations based on the interaction between climate change and social determinants of health. People who are more likely to experience adverse health outcomes as a result of underlying social and economic factors are often those most impacted by environmental hazards, including those resulting from climate change [1]. These populations are not only limited in their ability to recover from the growing number of natural disasters and extreme weather events, but may also be at greater risk of chronic conditions, food insecurity and subsequent malnutrition, and poor living conditions as a result of displacement, all of which can be predisposing factors for fungal infections [1,38].

Expanded surveillance, environmental sampling, and molecular analyses are critical to better understand the potential effects of climate change on the spatiotemporal trends of mycotic diseases and offer insights into the emergence of new fungal pathogens. The heavy interdependence of fungi and their surrounding ecosystems underscores the importance of recognizing the possibility of both direct and indirect impacts of climate change on fungal infections. Further exploration to assess the potential alterations to fungal pathogens and their impact on human disease caused by changes in the environment is essential to increase awareness and inform public health action.

## References

[1] Fifth National Climate Assessment [Internet]. [cited 2023 Dec 4]. https://nca2023.globalchange.gov/.

[2] Nnadi NE, Carter DA. Climate change and the emergence of fungal pathogens. PLoS Pathog. 2021 Apr 29;17(4):e1009503. doi: 10.1371/journal.ppat.1009503 33914854 PMC8084208

[3] Verweij PE, Arendrup MC, Alastruey-Izquierdo A, Gold JAW, Lockhart SR, Chiller T, et al. Dual use of antifungals in medicine and agriculture: How do we help prevent resistance developing in human pathogens? Drug Resist Updat. 2022 Dec;65:100885. doi: 10.1016/j.drup.2022.100885 36283187 PMC10693676

[4] Head JR, Sondermeyer-Cooksey G, Heaney AK, Yu AT, Jones I, Bhattachan A, et al. Effects of precipitation, heat, and drought on incidence and expansion of coccidioidomycosis in western USA: a longitudinal surveillance study. Lancet Planet Health. 2022 Oct 1;6(10):e793–e803. doi: 10.1016/S2542-5196(22)00202-9 36208642 PMC10189771

[5] Oltean HN, Etienne KA, Roe CC, Gade L, McCotter OZ, Engelthaler DM, et al. Utility of Whole-Genome Sequencing to Ascertain Locally Acquired Cases of Coccidioidomycosis, Washington, USA. Emerg Infect Dis. 2019 Mar;25(3):501–506. doi: 10.3201/eid2503.181155 30789132 PMC6390764

[6] Gorris ME, Treseder KK, Zender CS, Randerson JT. Expansion of Coccidioidomycosis Endemic Regions in the United States in Response to Climate Change. GeoHealth. 2019;3(10):308–327. doi: 10.1029/2019GH000209 32159021 PMC7007157

[7] Maiga AW, Deppen S, Scaffidi BK, Baddley J, Aldrich MC, Dittus RS, et al. Mapping Histoplasma capsulatum Exposure, United States. Emerg Infect Dis. 2018 Oct;24(10):1835–1839. doi: 10.3201/eid2410.180032 30226187 PMC6154167

[8] Mazi PB, Sahrmann JM, Olsen MA, Coler-Reilly A, Rauseo AM, Pullen M, et al. The Geographic Distribution of Dimorphic Mycoses in the United States for the Modern Era. Clin Infect Dis. 2023 Apr 1;76(7):1295–1301. doi: 10.1093/cid/ciac882 36366776 PMC10319749

[9] Byrnes EJ, Bildfell R, Frank SA, Mitchell TG, Marr K, Heitman J. Molecular Evidence that the Vancouver Island Cryptococcus gattii Outbreak has Expanded into the United States Pacific Northwest. J Infect Dis. 2009 Apr 1;199(7):1081–1086. 19220140 10.1086/597306 PMC2715219

[10] Cogliati M. Global warming impact on the expansion of fundamental niche of Cryptococcus gattii VGI in Europe. Environ Microbiol Rep. 2021 Jun;13(3):375. doi: 10.1111/1758-2229.12945 33945219 PMC8251527

[11] McKinsey DS, Pappas PG. Histoplasmosis: Time to Redraw the Map and Up Our Game. Clin Infect Dis. 2020 Mar 3;70(6):1011–1013. doi: 10.1093/cid/ciz327 31038169

[12] Taylor JW, Barker BM. The endozoan, small-mammal reservoir hypothesis and the life cycle of Coccidioides species. Med Mycol. 2019 Feb;57(Suppl 1):S16–S20. doi: 10.1093/mmy/myy039 30690603 PMC6702415

[13] Salazar-Hamm PS, Montoya KN, Montoya L, Cook K, Liphardt S, Taylor JW, et al. Breathing can be dangerous: Opportunistic fungal pathogens and the diverse community of the small mammal lung mycobiome. Front Fungal Biol. 2022 [cited 2024 Feb 6];3. Available from: https://www.frontiersin.org/articles/ doi: 10.3389/ffunb.2022.996574 37746221 PMC10512277

[14] Benedict K, Park BJ. Invasive Fungal Infections after Natural Disasters. Emerg Infect Dis. 2014 Mar;20(3):349–355. doi: 10.3201/eid2003.131230 24565446 PMC3944874

[15] Neblett Fanfair R, Benedict K, Bos J, Bennett SD, Lo YC, Adebanjo T, et al. Necrotizing Cutaneous Mucormycosis after a Tornado in Joplin, Missouri, in 2011. N Engl J Med. 2012 Dec 6;367(23):2214–2225. doi: 10.1056/NEJMoa1204781 23215557

[16] Tong DQ, Wang JXL, Gill TE, Lei H, Wang B. Intensified dust storm activity and Valley fever infection in the southwestern United States. Geophys Res Lett. 2017;44(9):4304–4312. doi: 10.1002/2017GL073524 30166741 PMC6108409

[17] Tong DQ, Gorris ME, Gill TE, Ardon-Dryer K, Wang J. Ren L. Dust Storms, Valley Fever, and Public Awareness. GeoHealth. 2022;6(8):e2022GH000642. 10.1029/2022GH000642 PMC9356325 35949254

[18] Basic Facts about Mold and Dampness. Mold. CDC; 2022 [cited 2023 Jan 6]. https://www.cdc.gov/mold/faqs.htm.

[19] Toda M, Williams S, Jackson BR, Wurster S, Serpa JA, Nigo M, et al. Invasive mold infections following Hurricane Harvey—Houston, Texas. Open Forum Infect Dis. 2023 Feb 21:ofad093. doi: 10.1093/ofid/ofad093 36910694 PMC10003735

[20] Kontoyiannis DP, Shah EC, Wurster S, Aitken SL, Graviss L, Raad II, et al. Culture-Documented Invasive Mold Infections at MD Anderson Cancer Center in Houston, Texas, Pre–and Post–Hurricane Harvey. Open Forum Infect Dis. 2019 Apr 1;6(4):ofz138. doi: 10.1093/ofid/ofz138 31024975 PMC6475585

[21] Wurster S, Paraskevopoulos T, Toda M, Jiang Y, Tarrand JJ, Williams S, et al. Invasive mould infections in patients from floodwater-damaged areas after hurricane Harvey–a closer look at an immunocompromised cancer patient population. J Infect. 2022 Mar. 12 [cited 2022 Apr 19]. Available from: https://www.sciencedirect.com/science/article/pii/S0163445322001359. 10.1016/j.jinf.2022.03.009 PMC11018252 35288118

[22] Benedict K, Jackson BR, Toda M. Diagnosis codes for mold infections and mold exposure before and after Hurricane Harvey among a commercially insured population-Houston, Texas, 2016–2018. Disaster Med Public Health Prep. 2023 Mar;17:1–10. 10.1017/dmp.2023.28 PMC10640901 36927602

[23] Dogra S, Uprety S. The menace of chronic and recurrent dermatophytosis in India: Is the problem deeper than we perceive? Indian Dermatol Online J. 2016;7(2):73–76. doi: 10.4103/2229-5178.178100 27057485 PMC4804598

[24] Uhrlaß S, Verma SB, Gräser Y, Rezaei-Matehkolaei A, Hatami M, Schaller M, et al. Trichophyton indotineae—An Emerging Pathogen Causing Recalcitrant Dermatophytoses in India and Worldwide—A Multidimensional Perspective. J Fungi. 2022 Jul;8(7):757. doi: 10.3390/jof8070757 35887512 PMC9323571

[25] Köhler JR, Casadevall A, Perfect J. The Spectrum of Fungi That Infects Humans. Cold Spring Harb Perspect Med. 2015 Jan;5(1):a019273. 10.1101/cshperspect.a019273 PMC4292074 25367975

[26] Robert VA, Casadevall A. Vertebrate Endothermy Restricts Most Fungi as Potential Pathogens. J Infect Dis. 2009 Nov 15;200(10):1623–1626. doi: 10.1086/644642 19827944

[27] Garcia-Solache MA, Casadevall A. Global Warming Will Bring New Fungal Diseases for Mammals. mBio. 2010 May 18;1(1). doi: 10.1128/mBio.00061-10 20689745 PMC2912667

[28] Casadevall A, Kontoyiannis DP, Robert V. On the Emergence of Candida auris: Climate Change, Azoles, Swamps, and Birds. Kronstad JW, editor. mBio. 2019 Aug 27;10(4):e01397–19. doi: 10.1128/mBio.01397-19 31337723 PMC6650554

[29] Gusa A, Yadav V, Roth C, Williams JD, Shouse EM, Magwene P, et al. Genome-wide analysis of heat stress-stimulated transposon mobility in the human fungal pathogen Cryptococcus deneoformans. Proc Natl Acad Sci U S A. 2023 Jan 24;120(4):e2209831120. doi: 10.1073/pnas.2209831120 36669112 PMC9942834

[30] Gusa A, Williams JD, Cho JE, Averette AF, Sun S, Shouse EM, et al. Transposon mobilization in the human fungal pathogen Cryptococcus is mutagenic during infection and promotes drug resistance in vitro. Proc Natl Acad Sci U S A. 2020 May 5;117(18):9973–9980. doi: 10.1073/pnas.2001451117 32303657 PMC7211991

[31] Toda M, Beer KD, Kuivila KM, Chiller TM, Jackson BR. Trends in Agricultural Triazole Fungicide Use in the United States, 1992–2016 and Possible Implications for Antifungal-Resistant Fungi in Human Disease. Environ Health Perspect. 2021 May;129(5):55001. doi: 10.1289/EHP7484 33949891 PMC8098123

[32] Velásquez AC, Castroverde CDM, He SY. Plant-Pathogen Warfare under Changing Climate Conditions. Curr Biol. 2018 May 21;28(10):R619–R634. doi: 10.1016/j.cub.2018.03.054 29787730 PMC5967643

[33] Delgado-Baquerizo M, Guerra CA, Cano-Díaz C, Egidi E, Wang JT, Eisenhauer N, et al. The proportion of soil-borne pathogens increases with warming at the global scale. Nat Clim Chang. 2020 Jun;10(6):550–554.

[34] Rhodes LA, McCarl BA. An Analysis of Climate Impacts on Herbicide, Insecticide, and Fungicide Expenditures. Agronomy. 2020 May;10(5):745.

[35] van Rhijn N, Storer ISR, Birch M, Oliver JD, Bottery MJ, Bromley MJ. Aspergillus fumigatus strains that evolve resistance to the agrochemical fungicide ipflufenoquin in vitro are also resistant to olorofim. Nat Microbiol. 2024 Jan;9(1):29–34. doi: 10.1038/s41564-023-01542-4 38151646 PMC10769868

[36] Chariyalertsak S, Sirisanthana T, Supparatpinyo K, Nelson KE. Seasonal Variation of Disseminated Penicillium marneffei Infections in Northern Thailand: A Clue to the Reservoir? J Infect Dis. 1996 Jun 1;173(6):1490–1493. doi: 10.1093/infdis/173.6.1490 8648227

[37] Le T, Wolbers M, Chi NH, Quang VM, Chinh NT, Huong Lan NP, et al. Epidemiology, Seasonality, and Predictors of Outcome of AIDS-Associated Penicillium marneffei Infection in Ho Chi Minh City, Viet Nam. Clin Infect Dis. 2011 Apr 1;52(7):945–952. doi: 10.1093/cid/cir028 21427403 PMC3106230

[38] Jenks JD, Prattes J, Wurster S, Sprute R, Seidel D, Oliverio M, et al. Social determinants of health as drivers of fungal disease. eClinicalMedicine. 2023 Dec;66:102325. doi: 10.1016/j.eclinm.2023.102325 38053535 PMC10694587

